# Supplementary material for: Precision game engineering through reshaping strategic payoffs
Source: Sci Rep. 2024 Oct 24;14:25226. doi: 10.1038/s41598-024-72543-4 (PMC11502784; doi:10.1038/s41598-024-72543-4)
Supplement: Supplementary file 5 — Supplementary Legends. [file 41598_2024_72543_MOESM5_ESM.docx]

## Supplementary information

**Supplementary Figure S1**. The comprehensive list of solutions for the two-player game with a 5-by-5 payoff matrix.

**Supplementary Table S1**. CPU runtimes for identifying engineering interventions in games of varying complexity (**Fig. 6** data).

**Supplementary File 1**. Sample implementations of prototype games presented in this article as Google Colaboratory notebooks.

**Supplementary File 2**. The comprehensive list of the identified engineering strategies for the four-player game.
