# Supplementary material for: Precision game engineering through reshaping strategic payoffs
Source: Sci Rep. 2024 Oct 24;14:25226. doi: 10.1038/s41598-024-72543-4 (PMC11502784; doi:10.1038/s41598-024-72543-4)
Supplement: Supplementary file 1 — Supplementary Figure S1. [file 41598_2024_72543_MOESM1_ESM.pdf]

Player 2

Player 1

Solution 1

	S1	S2	S3	S4	S5
S1	(-4, -14)	(-3, -13)	(-15, -13)	(-15, -1)	(-14, -15)
S2	(-11, -9)	(0, -5)	(-12, -4)	(0, -13)	(-5, -12)
S3	(-12, -9)	(-11, -6)	(0, 0)	(-5, -9)	(-6, -5)
S4	(-5, -7)	(-4, -6)	(-9, -1)	(-6, -4)	(-1, -5)
S5	(-13, -10)	(-7 + 7.01, -8 + 7.01)	(-3 + 3.01, -10)	(-5, -1)	(-7, -14)

Solution 2

	S1	S2	S3	S4	S5
S1	(-4, -14)	(-3, -13)	(-15, -13)	(-15, -1)	(-14, -15)
S2	(-11, -9)	(0 + -3.0, -5)	(-12, -4)	(0, -13)	(-5, -12)
S3	(-12, -9)	(-11, -6)	(0, 0)	(-5, -9)	(-6, -5)
S4	(-5, -7)	(-4, -6)	(-9, -1)	(-6, -4)	(-1, -5)
S5	(-13, -10)	(-7 + 4.01, -8)	(-3 + 3.01, -10)	(-5, -1 + -7.01)	(-7, -14)

Solution 3

	S1	S2	S3	S4	S5
S1	(-4, -14)	(-3, -13)	(-15, -13)	(-15, -1)	(-14, -15)
S2	(-11, -9)	(0, -5)	(-12, -4)	(0, -13)	(-5, -12)
S3	(-12, -9)	(-11, -6)	(0, 0)	(-5, -9)	(-6, -5)
S4	(-5, -7)	(-4, -6)	(-9, -1)	(-6, -4)	(-1, -5)
S5	(-13, -10)	(-7 + 7.01, -8)	(-3 + 3.01, -10)	(-5, -1 + -7.01)	(-7, -14)

Solution 4

	S1	S2	S3	S4	S5
S1	(-4, -14)	(-3, -13)	(-15, -13)	(-15, -1)	(-14, -15)
S2	(-11, -9)	(0 + -0.01, -5)	(-12, -4)	(0, -13)	(-5, -12)
S3	(-12, -9)	(-11, -6)	(0, 0)	(-5, -9)	(-6, -5 + 5.01)
S4	(-5, -7)	(-4, -6)	(-9, -1)	(-6, -4)	(-1, -5)
S5	(-13, -10)	(-7 + 7.0, -8 + 7.01)	(-3, -10)	(-5, -1)	(-7, -14)

Solution 5

	S1	S2	S3	S4	S5
S1	(-4, -14)	(-3, -13)	(-15, -13)	(-15, -1)	(-14, -15)
S2	(-11, -9)	(0 + -3.0, -5)	(-12, -4)	(0, -13)	(-5, -12)
S3	(-12, -9)	(-11, -6)	(0, 0)	(-5, -9)	(-6, -5 + 5.01)
S4	(-5, -7)	(-4, -6)	(-9, -1)	(-6, -4)	(-1, -5)
S5	(-13, -10)	(-7 + 4.01, -8)	(-3, -10)	(-5, -1 + -7.01)	(-7, -14)

Solution 6

	S1	S2	S3	S4	S5
S1	(-4, -14)	(-3, -13)	(-15, -13)	(-15, -1)	(-14, -15)
S2	(-11, -9)	(0, -5)	(-12, -4)	(0, -13)	(-5, -12)
S3	(-12, -9)	(-11, -6)	(0, 0)	(-5, -9)	(-6, -5 + 5.01)
S4	(-5, -7)	(-4, -6)	(-9, -1)	(-6, -4)	(-1, -5)
S5	(-13, -10)	(-7 + 7.01, -8 + 7.01)	(-3, -10)	(-5, -1)	(-7, -14)

Solution 7

	S1	S2	S3	S4	S5
S1	(-4, -14)	(-3, -13)	(-15, -13)	(-15, -1)	(-14, -15)
S2	(-11, -9)	(0, -5)	(-12, -4)	(0, -13)	(-5, -12)
S3	(-12, -9)	(-11, -6)	(0, 0)	(-5, -9)	(-6, -5 + 5.01)
S4	(-5, -7)	(-4, -6)	(-9, -1)	(-6, -4)	(-1, -5)
S5	(-13, -10)	(-7 + 7.01, -8)	(-3, -10)	(-5, -1 + -7.01)	(-7, -14)

Solution 8

	S1	S2	S3	S4	S5
S1	(-4, -14)	(-3, -13)	(-15, -13)	(-15, -1)	(-14, -15)
S2	(-11, -9)	(0 + -3.0, -5)	(-12, -4)	(0, -13)	(-5, -12)
S3	(-12, -9)	(-11, -6 + 6.01)	(0, 0)	(-5, -9)	(-6, -5)
S4	(-5, -7)	(-4, -6)	(-9, -1)	(-6, -4)	(-1, -5)
S5	(-13, -10)	(-7 + 4.01, -8)	(-3, -10)	(-5, -1 + -7.01)	(-7, -14)

Solution 9

	S1	S2	S3	S4	S5
S1	(-4, -14)	(-3, -13)	(-15, -13)	(-15, -1)	(-14, -15)
S2	(-11, -9)	(0, -5)	(-12, -4)	(0, -13)	(-5, -12)
S3	(-12, -9)	(-11, -6 + 6.01)	(0, 0)	(-5, -9)	(-6, -5)
S4	(-5, -7)	(-4, -6)	(-9, -1)	(-6, -4)	(-1, -5)
S5	(-13, -10)	(-7 + 7.01, -8)	(-3, -10)	(-5, -1 + -7.01)	(-7, -14)

Solution 10

	S1	S2	S3	S4	S5
S1	(-4, -14)	(-3, -13)	(-15, -13)	(-15, -1)	(-14, -15)
S2	(-11, -9)	(0, -5)	(-12, -4)	(0, -13)	(-5, -12)
S3	(-12, -9)	(-11, -6 + 6.01)	(0, 0)	(-5, -9)	(-6, -5)
S4	(-5, -7)	(-4, -6)	(-9, -1)	(-6, -4)	(-1, -5)
S5	(-13, -10)	(-7 + 7.01, -8 + 7.01)	(-3, -10)	(-5, -1)	(-7, -14)

Solution 11

	S1	S2	S3	S4	S5
S1	(-4, -14)	(-3, -13)	(-15, -13)	(-15, -1)	(-14, -15)
S2	(-11, -9)	(0, -5)	(-12, -4)	(0, -13)	(-5, -12)
S3	(-12, -9)	(-11, -6)	(0, 0)	(-5, -9)	(-6, -5)
S4	(-5, -7)	(-4, -6)	(-9 + 9.01, -1)	(-6, -4)	(-1, -5)
S5	(-13, -10)	(-7 + 7.01, -8)	(-3, -10)	(-5, -1 + -7.01)	(-7, -14)

Solution 12

	S1	S2	S3	S4	S5
S1	(-4, -14)	(-3, -13)	(-15, -13)	(-15, -1)	(-14, -15)
S2	(-11, -9)	(0, -5)	(-12, -4)	(0, -13)	(-5, -12)
S3	(-12, -9)	(-11, -6)	(0, 0)	(-5, -9)	(-6, -5)
S4	(-5, -7)	(-4, -6)	(-9 + 9.01, -1)	(-6, -4)	(-1, -5)
S5	(-13, -10)	(-7 + 7.01, -8 + 7.01)	(-3, -10)	(-5, -1)	(-7, -14)

Solution 13

	S1	S2	S3	S4	S5
S1	(-4, -14)	(-3, -13)	(-15, -13)	(-15, -1)	(-14, -15)
S2	(-11, -9)	(0, -5)	(-12, -4)	(0, -13)	(-5, -12)
S3	(-12, -9 + 9.01)	(-11, -6)	(0, 0)	(-5, -9)	(-6, -5)
S4	(-5, -7)	(-4, -6)	(-9, -1)	(-6, -4)	(-1, -5)
S5	(-13, -10)	(-7 + 7.01, -8)	(-3, -10)	(-5, -1 + -7.01)	(-7, -14)

Solution 14

	S1	S2	S3	S4	S5
S1	(-4, -14)	(-3, -13)	(-15, -13)	(-15, -1)	(-14, -15)
S2	(-11, -9)	(0, -5)	(-12, -4)	(0, -13)	(-5, -12)
S3	(-12, -9)	(-11, -6)	(0, 0)	(-5, -9 + 9.01)	(-6, -5)
S4	(-5, -7)	(-4, -6)	(-9, -1)	(-6, -4)	(-1, -5)
S5	(-13, -10)	(-7 + 7.01, -8)	(-3, -10)	(-5, -1 + -7.01)	(-7, -14)

Solution 15

	S1	S2	S3	S4	S5
S1	(-4, -14)	(-3, -13)	(-15, -13)	(-15, -1)	(-14, -15)
S2	(-11, -9)	(0, -5)	(-12, -4)	(0, -13)	(-5, -12)
S3	(-12, -9 + 9.01)	(-11, -6)	(0, 0)	(-5, -9)	(-6, -5)
S4	(-5, -7)	(-4, -6)	(-9, -1)	(-6, -4)	(-1, -5)
S5	(-13, -10)	(-7 + 7.01, -8 + 7.01)	(-3, -10)	(-5, -1)	(-7, -14)

Solution 16

	S1	S2	S3	S4	S5
S1	(-4, -14)	(-3, -13)	(-15, -13)	(-15, -1)	(-14, -15)
S2	(-11, -9)	(0, -5)	(-12, -4)	(0, -13)	(-5, -12)
S3	(-12, -9)	(-11, -6)	(0, 0)	(-5, -9 + 9.01)	(-6, -5)
S4	(-5, -7)	(-4, -6)	(-9, -1)	(-6, -4)	(-1, -5)
S5	(-13, -10)	(-7 + 7.01, -8 + 7.01)	(-3, -10)	(-5, -1)	(-7, -14)

Solution 17

	S1	S2	S3	S4	S5
S1	(-4, -14)	(-3 + -4.01, -13)	(-15, -13)	(-15, -1)	(-14, -15)
S2	(-11, -9)	(0 + -7.01, -5)	(-12, -4)	(0, -13)	(-5, -12)
S3	(-12, -9)	(-11, -6)	(0, 0)	(-5, -9)	(-6, -5)
S4	(-5, -7)	(-4 + -3.01, -6)	(-9, -1)	(-6, -4)	(-1, -5)
S5	(-13, -10)	(-7, -8)	(-3 + 3.01, -10)	(-5, -1 + -7.01)	(-7, -14)

Solution 18

	S1	S2	S3	S4	S5
S1	(-4, -14)	(-3 + -4.01, -13)	(-15, -13)	(-15, -1)	(-14, -15)
S2	(-11, -9)	(0 + -7.01, -5)	(-12, -4)	(0, -13)	(-5, -12)
S3	(-12, -9)	(-11, -6)	(0, 0)	(-5, -9)	(-6, -5)
S4	(-5, -7)	(-4 + -3.01, -6)	(-9, -1)	(-6, -4)	(-1, -5)
S5	(-13, -10)	(-7, -8 + 7.01)	(-3 + 3.01, -10)	(-5, -1)	(-7, -14)

Solution 19

	S1	S2	S3	S4	S5
S1	(-4, -14)	(-3, -13)	(-15, -13)	(-15, -1)	(-14, -15)
S2	(-11, -9)	(0 + -3.0, -5)	(-12 + 12.01, -4)	(0, -13)	(-5, -12)
S3	(-12, -9)	(-11, -6)	(0, 0)	(-5, -9)	(-6, -5)
S4	(-5, -7)	(-4, -6)	(-9, -1)	(-6, -4)	(-1, -5)
S5	(-13, -10)	(-7 + 4.01, -8)	(-3, -10)	(-5, -1 + -7.01)	(-7, -14)

Solution 20

	S1	S2	S3	S4	S5
S1	(-4, -14)	(-3, -13)	(-15, -13)	(-15, -1)	(-14, -15)
S2	(-11, -9)	(0, -5)	(-12 + 12.01, -4)	(0, -13)	(-5, -12)
S3	(-12, -9)	(-11, -6)	(0, 0)	(-5, -9)	(-6, -5)
S4	(-5, -7)	(-4, -6)	(-9, -1)	(-6, -4)	(-1, -5)
S5	(-13, -10)	(-7 + 7.01, -8)	(-3, -10)	(-5, -1 + -7.01)	(-7, -14)

Solution 21

	S1	S2	S3	S4	S5
S1	(-4, -14)	(-3, -13)	(-15, -13)	(-15, -1)	(-14, -15)
S2	(-11, -9)	(0 + -3.0, -5)	(-12 + 12.01, -4)	(0, -13)	(-5, -12)
S3	(-12, -9)	(-11, -6)	(0, 0)	(-5, -9)	(-6, -5)
S4	(-5, -7)	(-4, -6)	(-9, -1)	(-6, -4)	(-1, -5)
S5	(-13, -10)	(-7 + 4.01, -8 + 7.01)	(-3, -10)	(-5, -1)	(-7, -14)

Solution 22

	S1	S2	S3	S4	S5
S1	(-4, -14)	(-3, -13)	(-15, -13)	(-15, -1)	(-14, -15)
S2	(-11, -9)	(0, -5)	(-12 + 12.01, -4)	(0, -13)	(-5, -12)
S3	(-12, -9)	(-11, -6)	(0, 0)	(-5, -9)	(-6, -5)
S4	(-5, -7)	(-4, -6)	(-9, -1)	(-6, -4)	(-1, -5)
S5	(-13, -10)	(-7 + 7.01, -8 + 7.01)	(-3, -10)	(-5, -1)	(-7, -14)

Solution 23

	S1	S2	S3	S4	S5
S1	(-4, -14)	(-3 + -4.01, -13)	(-15, -13)	(-15, -1)	(-14, -15)
S2	(-11, -9)	(0 + -7.01, -5)	(-12, -4)	(0, -13)	(-5, -12)
S3	(-12, -9)	(-11, -6)	(0, 0)	(-5, -9)	(-6, -5 + 5.01)
S4	(-5, -7)	(-4 + -3.01, -6)	(-9, -1)	(-6, -4)	(-1, -5)
S5	(-13, -10)	(-7, -8 + 7.01)	(-3, -10)	(-5, -1)	(-7, -14)

Solution 24

	S1	S2	S3	S4	S5
S1	(-4, -14)	(-3 + -4.01, -13)	(-15, -13)	(-15, -1)	(-14, -15)
S2	(-11, -9)	(0 + -7.01, -5)	(-12, -4)	(0, -13)	(-5, -12)
S3	(-12, -9)	(-11, -6)	(0, 0)	(-5, -9)	(-6, -5 + 5.01)
S4	(-5, -7)	(-4 + -3.01, -6)	(-9, -1)	(-6, -4)	(-1, -5)
S5	(-13, -10)	(-7, -8)	(-3, -10)	(-5, -1 + -7.01)	(-7, -14)

Solution 25

	S1	S2	S3	S4	S5
S1	(-4, -14)	(-3 + -4.01, -13)	(-15, -13)	(-15, -1)	(-14, -15)
S2	(-11, -9)	(0 + -7.01, -5)	(-12, -4)	(0, -13)	(-5, -12)
S3	(-12, -9)	(-11, -6 + 6.01)	(0, 0)	(-5, -9)	(-6, -5)
S4	(-5, -7)	(-4 + -3.01, -6)	(-9, -1)	(-6, -4)	(-1, -5)
S5	(-13, -10)	(-7, -8)	(-3, -10)	(-5, -1 + -7.01)	(-7, -14)

Solution 26

	S1	S2	S3	S4	S5
S1	(-4, -14)	(-3 + -4.01, -13)	(-15, -13)	(-15, -1)	(-14, -15)
S2	(-11, -9)	(0 + -7.01, -5)	(-12, -4)	(0, -13)	(-5, -12)
S3	(-12, -9)	(-11, -6 + 6.01)	(0, 0)	(-5, -9)	(-6, -5)
S4	(-5, -7)	(-4 + -3.01, -6)	(-9, -1)	(-6, -4)	(-1, -5)
S5	(-13, -10)	(-7, -8 + 7.01)	(-3, -10)	(-5, -1)	(-7, -14)

Solution 27

	S1	S2	S3	S4	S5
S1	(-4, -14)	(-3, -13)	(-15 + 15.01, -13)	(-15, -1)	(-14, -15)
S2	(-11, -9)	(0, -5)	(-12, -4)	(0, -13)	(-5, -12)
S3	(-12, -9)	(-11, -6)	(0, 0)	(-5, -9)	(-6, -5)
S4	(-5, -7)	(-4, -6)	(-9, -1)	(-6, -4)	(-1, -5)
S5	(-13, -10)	(-7 + 7.01, -8)	(-3, -10)	(-5, -1 + -7.01)	(-7, -14)

Solution 28

	S1	S2	S3	S4	S5
S1	(-4, -14)	(-3, -13)	(-15 + 15.01, -13)	(-15, -1)	(-14, -15)
S2	(-11, -9)	(0, -5)	(-12, -4)	(0, -13)	(-5, -12)
S3	(-12, -9)	(-11, -6)	(0, 0)	(-5, -9)	(-6, -5)
S4	(-5, -7)	(-4, -6)	(-9, -1)	(-6, -4)	(-1, -5)
S5	(-13, -10)	(-7 + 7.01, -8 + 7.01)	(-3, -10)	(-5, -1)	(-7, -14)

Solution 29

	S1	S2	S3	S4	S5
S1	(-4, -14)	(-3 + -4.01, -13)	(-15, -13)	(-15, -1)	(-14, -15)
S2	(-11, -9)	(0 + -7.01, -5)	(-12, -4)	(0, -13)	(-5, -12)
S3	(-12, -9)	(-11, -6)	(0, 0)	(-5, -9 + 9.01)	(-6, -5)
S4	(-5, -7)	(-4 + -3.01, -6)	(-9, -1)	(-6, -4)	(-1, -5)
S5	(-13, -10)	(-7, -8)	(-3, -10)	(-5, -1 + -7.01)	(-7, -14)

Solution 30

	S1	S2	S3	S4	S5
S1	(-4, -14)	(-3 + -4.01, -13)	(-15, -13)	(-15, -1)	(-14, -15)
S2	(-11, -9)	(0 + -7.01, -5)	(-12, -4)	(0, -13)	(-5, -12)
S3	(-12, -9)	(-11, -6)	(0, 0)	(-5, -9 + 9.01)	(-6, -5)
S4	(-5, -7)	(-4 + -3.01, -6)	(-9, -1)	(-6, -4)	(-1, -5)
S5	(-13, -10)	(-7, -8 + 7.01)	(-3, -10)	(-5, -1)	(-7, -14)

Solution 31

	S1	S2	S3	S4	S5
S1	(-4, -14)	(-3 + -4.01, -13)	(-15, -13)	(-15, -1)	(-14, -15)
S2	(-11, -9)	(0 + -7.01, -5)	(-12, -4)	(0, -13)	(-5, -12)
S3	(-12, -9 + 9.01)	(-11, -6)	(0, 0)	(-5, -9)	(-6, -5)
S4	(-5, -7)	(-4 + -3.01, -6)	(-9, -1)	(-6, -4)	(-1, -5)
S5	(-13, -10)	(-7, -8)	(-3, -10)	(-5, -1 + -7.01)	(-7, -14)

Solution 32

	S1	S2	S3	S4	S5
S1	(-4, -14)	(-3 + -4.01, -13)	(-15, -13)	(-15, -1)	(-14, -15)
S2	(-11, -9)	(0 + -7.01, -5)	(-12, -4)	(0, -13)	(-5, -12)
S3	(-12, -9 + 9.01)	(-11, -6)	(0, 0)	(-5, -9)	(-6, -5)
S4	(-5, -7)	(-4 + -3.01, -6)	(-9, -1)	(-6, -4)	(-1, -5)
S5	(-13, -10)	(-7, -8 + 7.01)	(-3, -10)	(-5, -1)	(-7, -14)

Solution 33

	S1	S2	S3	S4	S5
S1	(-4, -14)	(-3 + -4.01, -13)	(-15, -13)	(-15, -1)	(-14, -15)
S2	(-11, -9)	(0 + -7.01, -5)	(-12, -4)	(0, -13)	(-5, -12)
S3	(-12, -9)	(-11, -6)	(0, 0)	(-5, -9)	(-6, -5)
S4	(-5, -7)	(-4 + -3.01, -6)	(-9 + 9.01, -1)	(-6, -4)	(-1, -5)
S5	(-13, -10)	(-7, -8)	(-3, -10)	(-5, -1 + -7.01)	(-7, -14)

Solution 34

	S1	S2	S3	S4	S5
S1	(-4, -14)	(-3 + -4.01, -13)	(-15, -13)	(-15, -1)	(-14, -15)
S2	(-11, -9)	(0 + -7.01, -5)	(-12, -4)	(0, -13)	(-5, -12)
S3	(-12, -9)	(-11, -6)	(0, 0)	(-5, -9)	(-6, -5)
S4	(-5, -7)	(-4 + -3.01, -6)	(-9 + 9.01, -1)	(-6, -4)	(-1, -5)
S5	(-13, -10)	(-7, -8 + 7.01)	(-3, -10)	(-5, -1)	(-7, -14)

Solution 35

	S1	S2	S3	S4	S5
S1	(-4, -14)	(-3 + -4.01, -13)	(-15, -13)	(-15, -1)	(-14, -15)
S2	(-11, -9)	(0 + -7.01, -5)	(-12 + 12.01, -4)	(0, -13)	(-5, -12)
S3	(-12, -9)	(-11, -6)	(0, 0)	(-5, -9)	(-6, -5)
S4	(-5, -7)	(-4 + -3.01, -6)	(-9, -1)	(-6, -4)	(-1, -5)
S5	(-13, -10)	(-7, -8 + 7.01)	(-3, -10)	(-5, -1)	(-7, -14)

Solution 36

	S1	S2	S3	S4	S5
S1	(-4, -14)	(-3 + -4.01, -13)	(-15, -13)	(-15, -1)	(-14, -15)
S2	(-11, -9)	(0 + -7.01, -5)	(-12 + 12.01, -4)	(0, -13)	(-5, -12)
S3	(-12, -9)	(-11, -6)	(0, 0)	(-5, -9)	(-6, -5)
S4	(-5, -7)	(-4 + -3.01, -6)	(-9, -1)	(-6, -4)	(-1, -5)
S5	(-13, -10)	(-7, -8)	(-3, -10)	(-5, -1 + -7.01)	(-7, -14)

Solution 37

	S1	S2	S3	S4	S5
S1	(-4, -14)	(-3 + -4.01, -13)	(-15 + 15.01, -13)	(-15, -1)	(-14, -15)
S2	(-11, -9)	(0 + -7.01, -5)	(-12, -4)	(0, -13)	(-5, -12)
S3	(-12, -9)	(-11, -6)	(0, 0)	(-5, -9)	(-6, -5)
S4	(-5, -7)	(-4 + -3.01, -6)	(-9, -1)	(-6, -4)	(-1, -5)
S5	(-13, -10)	(-7, -8 + 7.01)	(-3, -10)	(-5, -1)	(-7, -14)

Solution 38

	S1	S2	S3	S4	S5
S1	(-4, -14)	(-3 + -4.01, -13)	(-15 + 15.01, -13)	(-15, -1)	(-14, -15)
S2	(-11, -9)	(0 + -7.01, -5)	(-12, -4)	(0, -13)	(-5, -12)
S3	(-12, -9)	(-11, -6)	(0, 0)	(-5, -9)	(-6, -5)
S4	(-5, -7)	(-4 + -3.01, -6)	(-9, -1)	(-6, -4)	(-1, -5)
S5	(-13, -10)	(-7, -8)	(-3, -10)	(-5, -1 + -7.01)	(-7, -14)

Perturbed cells | Nash equilibrium

Supplementary Figure S1. The 38 unique ways of engineering a game with a 5-by-5 payoff matrix in order to change the NE from (*S*3, *S*3) to (*S*5, *S*2).
